# Annotations

**Published:** 1897-04-03

**Authors:** 


### Annotations.

### The Cause of Baldness.

Why men go bald has long been a puzzle. That it is in some sense an associate of senility is generally recognised, but that age is not a necessary factor in its production seems clearly demonstrated by the comparative youth of many of its victims. It has been attributed to interference with the circulation in the scalp by the pressure of tight or rigid hats, to over thinking, to under feeding, and to many other causes; and now, like so many other things, is attributed to a microbe. There could be no doubt, so far as all appearances went, that the condition in the bald and shiny patches of alopecia areata was the same as that which obtained in the general baldness. It became necessary, then, to find a similar cause for both conditions, and this is what has been done. According to a recent paper by Dr. Sabouraud, which appeared in the "Annales de l'Inst. Pasteur," the microbe of seborrhœa grows in the sebaceous glands, and when it affects hairy regions it causes the hair to fall out. The same cause is in operation both in general seborrhœa and in the circumscribed patches of alopecia areata. According to this view, then, alopecia areata is an acute localised form of seborrhœa, while baldness is a diffuse and widespread process of the same nature. All of which is very interesting, but, we need hardly say, does not go further than to tell us the mechanism by which baldness is produced. Why in some cases this microbe should grow into certain glands, and cause atrophy of the associated follicles and death of the hair, while other glands in neighbouring regions are left untouched, remains just as great a mystery as ever. Still, it is something to know that the actual destruction of the hair is due to a bacillus, for, even if we cannot stop the prime cause of its developing, we may perchance prevent the mischief it performs. That it is caused by microbic products is, at least, rendered probable by an experiment lately shown in Paris, where a rabbit was injected with the toxic liquids derived from cultures of the seborrhœa bacillus, with the result that all its hair fell off, without any other symptom occurring. Now for the anti-toxin and flowing locks!

### The Water in Public Baths.

The art of swimming is one of some utility; proficiency in it may be the means of saving life, and its practice ought to conduce to health, and no doubt sometimes does so. It is not unreasonable to suppose that the latter may depend on the character of the water in which the practice is indulged in, and it is much to be hoped that due attention is paid to the purity of the water supplied to the public swimming baths in this country. Professor Baginsky has recently been investigating the condition of the water in some of the public baths in Berlin, with the result that he found a very large increase in the number of bacteria after a considerable number of people had bathed. Thus, at one of the establishments, the water when recently admitted already contained many microbes, but whether these were present in the water before admission or were derived from the sides of the bath is not stated. After twelve hours, however, when 200 persons had used the bath, the water contained 90,000 germs per cubic centimetre; while after twenty-four hours, when 549 people had bathed, the germs amounted to 270,000 per cubic centimetre. The microbes found, it should be added, were of a distinctly unpleasant character, showing the filthy nature of the pollution from which they had arisen. The numbers given may seem large, but they do not represent a larger admixture with microbes than has been discovered in many an ice cream. All depends on their character, and it is impossible to deny that typhoid fever might be spread by this means, for it certainly is a by no means uncommon event for swimmers, especially learners, to swallow a gulp of the fluid in which they float. In this relation it should be mentioned that Professor Baginsky said he had seen several cases in which children had been attacked with typhoid fever after using the public baths. A penny swim is a very good and pleasant thing at certain times of the year, but it must be very difficult to insure that it shall be free from risk. As a mere matter of health and cleanliness, and all the year round utility, it may be questioned whether a penny spray bath ending with a shower is not a preferable arrangement for a working-class community. So far as infection is concerned, this ought to be capable of being made absolutely safe.

### The Venice Convention.

The fact that the International Convention, which has been drawn up by the Sanitary Conference of Venice, has been signed by so many of the Powers is a matter for much congratulation, for perhaps more than any of its predecessors does this convention embody the views of English sanitarians in regard to the proper mode of checking the spread of such diseases as cholera and plague. One great point for which England has always contended is that detention of the sick is sufficient, and that the healthy may be allowed to pass. Between us and some of the dwellers in the south of Europe there has, however, always been a difference which, perhaps, is not entirely unnatural. Another is that a country should be allowed to import merchandise from an infected place without thereby subjecting her commerce to restriction. In regard to both of these points the results of the conference are satisfactory. Shipping from British India will no longer be detained at Suez, if there is no plague on board, but will be escorted through the canal by guards and allowed to enter the Mediterranean, and any form of merchandise may be admitted by any country. The truth is that, strong as prejudice has always been upon this subject, facts have proved stronger still, and the sight of England remaining free from these diseases, notwithstanding the constant stream of passengers and the endless succession of cargoes of what would, a few years ago, have been called "infected" merchandise entering her ports, has been too much even for the most enthusiastic advocates of quarantine. A point on which far too little stress is laid by many who write on this subject is that transport by sea is but one of the ways by which these diseases are carried westwards. The old lines of transit by land are just as open now, and indeed far more open than they were hundreds of years ago, so that even if quarantine did all that has been claimed for it, it could but put off the evil day. No doubt the sea route is the quickest, but both plague and cholera have far more frequently come westwards over land than over sea.

